# Organ Transplants From Deceased Donors With Primary Brain Tumors and Risk of Cancer Transmission

**DOI:** 10.1001/jamasurg.2022.8419

**Published:** 2023-03-22

**Authors:** George H. B. Greenhall, Brian A. Rous, Matthew L. Robb, Chloe Brown, Gillian Hardman, Rachel M. Hilton, James M. Neuberger, John H. Dark, Rachel J. Johnson, John L. R. Forsythe, Laurie A. Tomlinson, Chris J. Callaghan, Christopher J. E. Watson

**Affiliations:** 1 Department of Statistics and Clinical Research, Organ and Tissue Donation and Transplantation Directorate, NHS Blood and Transplant, Bristol, United Kingdom; 2 School of Immunology and Microbial Sciences, King’s College London, London, United Kingdom; 3 National Cancer Registration and Analysis Service, Fulbourn, United Kingdom; 4 Translational and Clinical Research Institute, Faculty of Medical Sciences, Newcastle University, Newcastle, United Kingdom; 5 Department of Nephrology and Transplantation, Guy’s Hospital, London, United Kingdom; 6 Liver Unit, Queen Elizabeth Hospital NHS Foundation Trust, Birmingham, United Kingdom; 7 Department of Noncommunicable Disease Epidemiology, London School of Hygiene and Tropical Medicine, London, United Kingdom; 8 Department of Surgery, University of Cambridge, Cambridge, United Kingdom; 9 NIHR Blood and Transplant Research Unit in Organ Donation and Transplantation, University of Cambridge, Cambridge, United Kingdom

## Abstract

**Importance:**

Cancer transmission is a known risk for recipients of organ transplants. Many people wait a long time for a suitable transplant; some never receive one. Although patients with brain tumors may donate their organs, opinions vary on the risks involved.

**Objective:**

To determine the risk of cancer transmission associated with organ transplants from deceased donors with primary brain tumors. Key secondary objectives were to investigate the association that donor brain tumors have with organ usage and posttransplant survival.

**Design, Setting, and Participants:**

This was a cohort study in England and Scotland, conducted from January 1, 2000, to December 31, 2016, with follow-up to December 31, 2020. This study used linked data on deceased donors and solid organ transplant recipients with valid national patient identifier numbers from the UK Transplant Registry, the National Cancer Registration and Analysis Service (England), and the Scottish Cancer Registry. For secondary analyses, comparators were matched on factors that may influence the likelihood of organ usage or transplant failure. Statistical analysis of study data took place from October 1, 2021, to May 31, 2022.

**Exposures:**

A history of primary brain tumor in the organ donor, identified from all 3 data sources using disease codes.

**Main Outcomes and Measures:**

Transmission of brain tumor from the organ donor into the transplant recipient. Secondary outcomes were organ utilization (ie, transplant of an offered organ) and survival of kidney, liver, heart, and lung transplants and their recipients. Key covariates in donors with brain tumors were tumor grade and treatment history.

**Results:**

This study included a total of 282 donors (median [IQR] age, 42 [33-54] years; 154 females [55%]) with primary brain tumors and 887 transplants from them, 778 (88%) of which were analyzed for the primary outcome. There were 262 transplants from donors with high-grade tumors and 494 from donors with prior neurosurgical intervention or radiotherapy. Median (IQR) recipient age was 48 (35-58) years, and 476 (61%) were male. Among 83 posttransplant malignancies (excluding NMSC) that occurred over a median (IQR) of 6 (3-9) years in 79 recipients of transplants from donors with brain tumors, none were of a histological type matching the donor brain tumor. Transplant survival was equivalent to that of matched controls. Kidney, liver, and lung utilization were lower in donors with high-grade brain tumors compared with matched controls.

**Conclusions and Relevance:**

Results of this cohort study suggest that the risk of cancer transmission in transplants from deceased donors with primary brain tumors was lower than previously thought, even in the context of donors that are considered as higher risk. Long-term transplant outcomes are favorable. These results suggest that it may be possible to safely expand organ usage from this donor group.

## Key Points

**Question:**

What is the risk of cancer transmission from deceased donors with primary brain tumors to the recipients of their organs?

**Findings:**

In this national cohort study of 778 transplants from 282 deceased donors with primary brain tumors, including 262 transplants from donors with high-grade brain tumors, there were no cases of brain tumor transmission. Some organs from donors with high-grade tumors were less likely to be transplanted, and organ transplant survival was equivalent to that in matched controls.

**Meaning:**

Results suggest that the risk of cancer transmission in transplants from deceased donors with primary brain tumors is lower than previously thought; it may be possible to safely expand the use of organs from this donor group.

## Teaser

This cohort study investigates the risk of recipient cancer transmission associated with organ transplants from deceased donors with primary brain tumors.

## Introduction

Organ transplants carry an unavoidable but small risk of disease transmission from donor to recipient.^1,2,3,4^ Although active malignancy is usually a contraindication to organ donation, the use of organs from patients with primary brain tumors is generally accepted because these cancers rarely spread beyond the central nervous system.^5^ Therefore, though possible, the risk of cancer transmission appears to be lower than that of other tumors.^6^

Opinions vary on the safety of organ transplants from donors with brain tumors.^7,8,9^ Biased recording, limited size, and insufficient detail limit existing studies in this area, leading to conflicting risk estimates.^10,11,12,13,14,15,16,17^ Although higher tumor grade or a history of surgical intervention may increase the risk of transmission through systemic dissemination before organ donation, evidence supporting this is lacking or outdated.^18,19,20,21,22^ This uncertainty is reflected in international guidelines, in which risk stratification varies greatly, with guidance in the US being notably more conservative than in Europe.^5,23,24^

Despite global increases in deceased organ donation rates, each year thousands of people waiting for a transplant die or become too unwell to undergo the procedure.^25,26,27,28^ Underlying this is the ongoing shortfall of suitable organs for transplant recipients. Optimized organ utilization, which refers to maximizing the benefits of organ transplants while maintaining safety, is a key strategy for addressing this shortfall, and remains an international priority. This requires consideration of donors with conditions that may confer greater risk to transplant recipients, including potentially transmissible diseases.^29,30,31^ Because patients dying of brain tumors, who tend to be younger and otherwise well, may donate good-quality organs, greater utilization of such donors could benefit many patients waiting for a transplant.^5,23,24,32^ Better understanding of the risks and benefits in this context may help transplant clinicians and their patients, who often face difficult decisions when offered organs from higher risk donors.

We examined the experience of organ donation from deceased donors with primary brain tumors in the UK. We looked for evidence of cancer transmission to transplant recipients, compared long-term transplant survival, and studied the association with organ utilization.

## Methods

### Study Design

The study received approval from the NHS Health Research Authority London-Surrey Research Ethics Committee and the NHS Scotland Public Benefit and Privacy Panel for Health and Social Care. Individuals who opted out of their data being used for research by National Health Service Blood and Transplant (NHSBT) were excluded.

This was a cohort study using linked data from 3 sources. The UK Transplant Registry (UKTR) is managed by NHSBT as part of its legal duty to monitor the safety of the national organ transplant program in the UK.^33^ It holds data on donor characterization (including a history of cancer), recipient details, and long-term transplant outcomes; for kidney recipients, this includes details of cancers occurring after transplant.^34^ NHSBT routinely collects ethnicity data, but these were not used in this study. UK transplant centers must also report all suspected disease transmission events to NHSBT. NHSBT coordinates allocation of organs from deceased donors across the whole of the UK; all patients waiting for a transplant have an equal chance of being offered an organ from a donor with a primary brain tumor.^35^

The study population comprised all deceased donors and transplant recipients in England and Scotland between January 1, 2000, and December 31, 2016. UKTR data were linked to 2 national cancer registries with excellent coverage of a combined population of approximately 62 million people: the National Cancer Registration and Analysis Service (NCRAS) in England and the Scottish Cancer Registry (SCR) (eFigure 1 in Supplement 1).^36,37,38^ All donors and recipients with valid national patient identifier numbers were included.^39^ We followed the Strengthening the Reporting of Observational Studies in Epidemiology (STROBE) reporting guidelines.

### Case Identification

We found donors with primary brain tumors using *International Statistical Classification of Diseases and Related Health Problems, Tenth Revision (ICD-10)* codes in the cancer registries and cause of death codes in the UKTR (eTable 1 in Supplement 1).^40^ We included all types of primary brain tumor and excluded intracranial lymphoma (an absolute contraindication to organ donation in the UK), spinal cord tumors, and cranial nerve tumors.^41^

We mapped brain tumor grade according to the World Health Organization (WHO) 2007 system using *ICD for Oncology, Third Edition* (*ICD-O-3*) morphology codes with manual curation where necessary (eTable 2 in Supplement 1).^42,43^ In grouped analyses, the low-grade category included grades 1 and 2 and the high-grade category included grades 3 and 4. Where a definitive grade was not available, we categorized tumors as low, high, or unknown grade, based on the information recorded. For donors with more than 1 brain tumor, the neoplasm with the highest grade was the primary diagnosis. Where a tumor had progressed between diagnosis and donation, we used the most recent (ie, higher) grade and the original date of diagnosis. We included tumors with generic morphology (eg, “neoplasm, uncertain behavior”), unless UKTR records clearly showed an alternative diagnosis.

Treatment history data came from Operating Procedure Codes Supplement 4 (OPCS-4) codes (NCRAS data) (eTable 3 in Supplement 1),^44^ standardized treatment fields (SCR data), and manual review of UKTR records. This included radiotherapy, resection, biopsy, external ventricular drain insertion, and the presence of a cerebroventricular shunt but excluded procedures at the time of organ retrieval.

Where possible, we categorized tumors according to their transmission risk, using both US and UK guidelines. The US Organ Procurement and Transplantation Network (OPTN) Disease Transmission Advisory Committee classifies all low-grade tumors (ie, grade 1 or 2) as low risk (0.1%-1% transmission risk) and all high-grade tumors (ie, grade 3 or 4) or any tumor (regardless of grade) with previous radiotherapy or neurosurgery as high risk (>10%; although the guidelines state that tumors such as “uncomplicated glioblastoma” may be considered as intermediate risk [1-10%]).^23^ In the UK, the Advisory Committee on the Safety of Blood, Tissue and Organs (SaBTO) defines grade 3 tumors as lower risk (<2%) and grade 4 as intermediate risk (2.2%; upper 95% CI, 6.4%).^5^ Although not included in SaBTO guidelines, we considered low-grade brain tumors as having minimal transmission risk (<0.1%) in accordance with other noninvasive tumors.

Having identified deceased donors with primary brain tumors, we traced all recipients of resulting solid-organ transplants (kidney, liver, heart, lung, pancreas, bowel, or multiorgan transplants) within the study population. We used donors without brain tumors and the recipients of their organs as comparators in transplant survival and organ utilization analyses.

We summarized standard indicators of organ quality, including donor type (donation after circulatory death vs donation after brain death), body mass index (BMI), comorbidities, terminal serum creatinine (ie, the last result recorded before organ retrieval), and validated organ-specific risk indices (UK Kidney Donor Risk Index, UK Donor Liver Index [UK DLI], lung donor category).^45,46,47^

### Outcomes

#### Donor-Transmitted Cancer

The main outcome was cancer transmission. To detect cases, 3 authors (G.H.B.G., C.J.C., C.J.E.W.) reviewed all malignant tumors after transplant in recipients of transplants from donors with brain tumors, comparing them with their donor’s tumor. This used cancer registry diagnoses, UKTR follow-up data (in kidney recipients), and NHSBT clinical governance records, with expert review from a coauthor (B.A.R.) where necessary.

We excluded benign and in situ tumors and nonmelanoma skin cancer (NMSC). NCRAS data covered all recipient cancer diagnoses up to April 4, 2020, and SCR up to December 31, 2018, giving all recipients at least 2 years of follow-up for posttransplant cancer incidence.

#### Transplant Survival

The long-term outcome was transplant failure, a composite of death, repeat transplant or (in kidney recipients) resumption of long-term dialysis, censored at 10 years or last known follow-up in the UKTR. We restricted this analysis to 4 transplant types (kidney, liver, heart and lung, excluding multiorgan transplants). In sensitivity analyses, we compared death and graft failure separately.

This analysis used matched controls to account for clinical heterogeneity. For each transplant from a donor with a brain tumor, we selected 4 controls (transplants from donors without brain tumors) randomly from the study population, matched on factors that influence patient or graft survival, making a separate control group for each transplant type.^48^ All controls were matched on donor and recipient age (±10 years) and sex. Kidney transplants were additionally matched on donor type, terminal creatinine (</≥100 μmol/L; to convert serum creatinine to milligram per deciliter, divide by 88.4), graft number (primary vs other), and calendar period (2000-2009 and 2010-2016). Liver and heart transplants were additionally matched on donor type, calendar period, and wait-list urgency (patients with shorter life expectancy are prioritized for organ allocation according to national criteria).^49,50,51^ This analysis used UKTR data collected up to December 31, 2020, and excluded transplants if no follow-up data were available, matching variables were missing, or no controls could be matched.

#### Organ Utilization

This analysis included all consented donors, which are defined as individuals without an absolute contraindication to donation where consent (in England) or authorization (in Scotland) for organ donation has been granted,^41^ and was restricted to donors where at least 1 organ was offered for transplant. We first explored whether donor utilization (defined as the generation of at least 1 organ transplant) was associated with tumor grade or treatment history (resection, shunt, or radiotherapy).

We then compared organ-specific utilization rates (defined as the proportion of offered organs that were transplanted) between donors with brain tumors and those without. This analysis used control donors matched on factors that may influence organ utilization (sex, age, type, terminal creatinine, hypertension, smoking and calendar period) in a 4:1 ratio, stratified by tumor grade (high/low, with controls matched separately). Donors with brain tumors of unknown grade or those with incomplete data on matching variables were excluded. Where there was evidence of a difference in organ utilization between donors with brain tumors and matched controls, we estimated the additional number of each organ that would have been transplanted from donors with brain tumors if the utilization rates had been equivalent in the 2 groups.

### Statistical Analysis

We examined categorical variables using χ^2^ tests and transplant survival with the Kaplan-Meier method. All analyses used SAS Enterprise Guide, version 7.13 (SAS Institute Inc). Statistical tests were conducted with a 2-sided significance level of 5%. Statistical analysis of study data took place from October 1, 2021, to May 31, 2022.

## Results

### Study Population

Among 13 274 solid organ donors in the study population, 282 (2%) had primary brain tumors. Median [IQR] age of donors with primary brain tumors was 42 (33-54) years; 154 (55%) were female, and 128 (45%) were male. Compared with donors without brain tumors, those with brain tumors were younger, had fewer comorbidities (eg, hypertension, 42 of 282 [15%] vs 3230 of 12 992 [25%]) and lifestyle risk factors (eg, smoking, 69 of 282 [24%] vs 5970 of 12 992 [46%]) and more favorable organ risk markers (eg, median [IQR] terminal creatinine, 65 [50-81] μmol/L vs 75 [59-97] μmol/L) (Table 1).^42^ Median (IQR) time from brain tumor diagnosis to death was 8 (2-463) days. A total of 210 of 282 tumors (74%) had a histological diagnosis, and 22 of 282 (8%) were confirmed on biopsy at the time of organ retrieval.

**Table 1.** Characteristics of Deceased Donors in the Study Population^a^

Characteristic	No. (%)	
	Donors with brain tumors (n = 282)	Donors without brain tumors (n = 12 992)
Donor characteristics		
Age, median (IQR), y	42 (33-54)	50 (37-60)
Female sex	154 (55)	6044 (47)
Male sex	128 (45)	6947 (53)
DCD	41 (15)	3744 (29)
BMI, median (IQR)^b^	26 (23-28)	25 (23-29)
Diabetes	10 (4)	773 (6)
Hypertension	42 (15)	3230 (25)
Smoking^c^	69 (24)	5970 (46)
Alcohol abuse^c^	10 (4)	1566 (12)
Drug abuse^c^	14 (5)	817 (6)
Organ-specific risk indices^d^		
Kidney		
Terminal creatinine, median (IQR), μmol/L	65 (50-81)	75 (59-97)
UK KDRI^45^^,^^e^	0.99 (0.81-1.30)	1.04 (0.96-1.46)
Liver		
UK DLI,^46^ median (IQR)	0.98 (0.85-1.12)	1.10 (0.94-1.33)
Lung donor category^47^		
Optimal	32 (51)	456 (23)
Extended criteria	14 (22)	891 (44)
Marginal	6 (10)	81 (4)
Tumor characteristics		
Time since diagnosis, median (IQR), d	8 (2-463)	NA
Histological diagnosis	210 (74)	
WHO grade^f^		
1	90 (32)	NA
2	49 (18)	
3	28 (9)	
4	54 (19)	
Grade uncertain		
Low grade	14 (5)^g^	NA
High grade	13 (5)^h^	
Grade unavailable	34 (12)^i^	
Treatment history		
Resection	140 (50)	NA
Cerebroventricular shunt	17 (6)	
Radiotherapy	29 (10)	

Abbreviations: BMI, body mass index; DCD, donation after circulatory death; DLI, Donor Liver Index; KDRI, Kidney Donor Risk Index; NA, not applicable; WHO, World Health Organization.

SI conversion factor: To convert serum creatinine to milligram per deciliter, divide by 88.4.

^a^ Missing data (n): sex (1), diabetes (353), hypertension (463), smoking (375), alcohol abuse (1598), drug abuse (480), BMI (333), terminal creatinine (234), UK KDRI (299), UK DLI (585), lung donor category (601), basis of diagnosis (4), time since diagnosis (6).

^b^ Calculated as weight in kilograms divided by height in meters squared.

^c^ Past or current.

^d^ Where organ transplanted.

^e^ Excludes donors aged <18 years.^45^

^f^ 2007 Classification system.^42^

^g^ Includes 5 with histological diagnosis.

^h^ Includes 4 with histological diagnosis.

^i^ Includes 7 with histological diagnosis.

A definitive grade was available for 221 tumors (78%); among the remaining 61, 15 underwent histological examination (eTable 4 in Supplement 1). Overall, there were 153 donors (53%) with low-grade brain tumors, 95 (34%) with high-grade tumors, and 34 (12%) with tumors of unknown grade. In total, 202 donors (72%) had undergone neurosurgical procedures before organ donation, including tumor resection (n = 140), external ventricular drain insertion (n = 51), shunt insertion (n = 17), and biopsy alone (n = 70); most procedures were performed in the month before donation. Twenty-nine donors (10%) had received radiotherapy (eTable 5 in Supplement 1). Based on OPTN criteria, 217 donors (77%) were high risk, and 39 (14%) were low risk (23 [8%] with glioblastoma multiforme and no history of neurosurgery or radiotherapy could be considered as “intermediate risk” by OPTN criteria).^23^ Using SaBTO groupings, 54 donors (19%) were intermediate risk, 28 (10%) were lower risk, and 153 (54%) were minimal risk (26 and 47 donors had insufficient data for OPTN and SABTO risk categorization, respectively).

**Figure 1.**
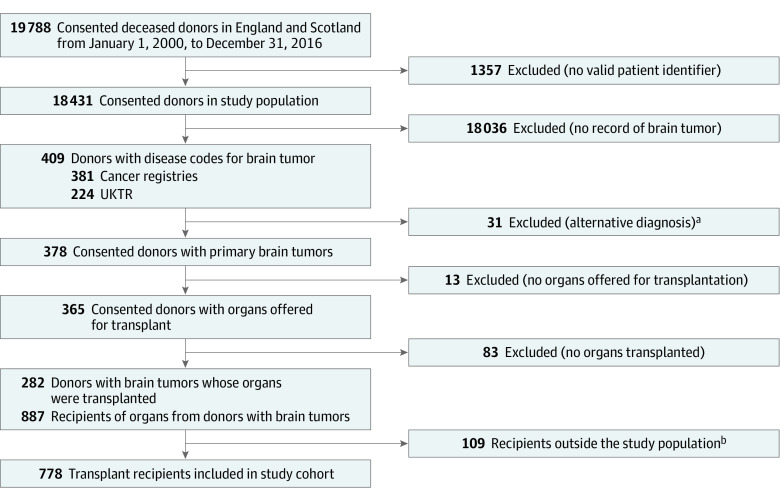
Cohort Creation Consented donors were individuals with no absolute contraindication, in whom consent/authorization for donation has been granted by the patient or their family. Donors were individuals from whom at least 1 solid organ was transplanted. Study population refers to the donors and recipients residing in England or Scotland with valid patient identifier numbers. UKTR indicates the UK Transplant Registry. ^a^Schwannoma (6), arteriovenous malformation (4), intracranial hemorrhage (3), cavernous hemangioma (2), dermoid cyst (2), colloid cyst (2), pituitary adenoma (2), cerebral abscess (1), cerebral infarction (1), clival chordoma (1), congenital malformation (1), Masson tumor (1), lymphoma (1), olfactory neuroblastoma (1), optic glioma (1), paraganglioma (1), and spinal ependymoma (1). ^b^Rest of UK (63), overseas (18), and England/Scotland without valid patient identifiers (28).

Donors with brain tumors gave 1014 organs to 887 recipients, of whom 778 (88%) were in the study population (the remaining 12% were in Wales, Northern Ireland, or overseas, or in England or Scotland without valid national patient identifier numbers) (Figure 1). There were 262 transplants from donors with high-grade brain tumors, including 81 and 142 from donors with grade 3 and 4 tumors, respectively (Table 2); 490 transplants (63%) came from donors with prior neurosurgical intervention or radiotherapy. Donors defined as high risk by OPTN guidelines generated 605 transplants (this includes 60 transplants from donors with glioblastoma and no history of neurosurgery or radiotherapy, which may be considered as “intermediate risk” by OPTN criteria). In the recipients of transplants from donors with brain tumors, median (IQR) age was 48 (35-58) years; 476 (61%) were male. Recipient characteristics were similar in transplants from donors with and without brain tumors (eTable 6 in Supplement 1). Median (IQR) recipient follow-up was 7 (4-11) years.

**Table 2.** Transplants From Deceased Donors With Brain Tumors Included in the Study Population

Transplant type	Donor brain tumor grade					Total
	Low^a^	High			Unknown	
		Grade 3	Grade 4	Uncertain^b^		
Kidney^c^	218	34	75	18	52	397
Liver	99	22	39	11	19	190
Heart	36	10	11	5	7	69
Lung	29	4	4	2	7	46
Kidney-pancreas	25	9	7	2	2	45
Other^d^	19	2	6	1	3	31
Total	426	81	142	39	90	778

^a^ Includes grade 1, grade 2, and low-grade tumor (1 or 2).

^b^ High-grade tumor (3 or 4).

^c^ Includes dual kidney transplants (8).

^d^ Pancreas alone (6), pancreas islets (7), heart-lung (5), heart-kidney (2), liver-kidney (3), multivisceral (3), modified multivisceral (3), bowel only (2).

### Donor-Transmitted Cancer

A total of 83 posttransplant malignancies (excluding NMSCs) occurred over a median (IQR) of 6 (3-9) years in 79 recipients of transplants from donors with brain tumors. Of 45 tumors in kidney recipients recorded in the cancer registries, 15 (33%) were reported to NHSBT. No recipient tumors had a histological type matching that of the donor brain tumor. There were 4 tumors in kidney recipients with unspecified primary site or histology, occurring between 4 and 14 years after transplant; expert review concluded that cancer transmission was highly unlikely in these cases. Transmission of donor brain tumors was excluded in all other cases. Aside from a renal cell carcinoma (pathologically distinct from the donor’s brain tumor), no cancer transmissions from the donors with brain tumors in our study were reported to NHSBT.

### Transplant Survival

**Figure 2.**
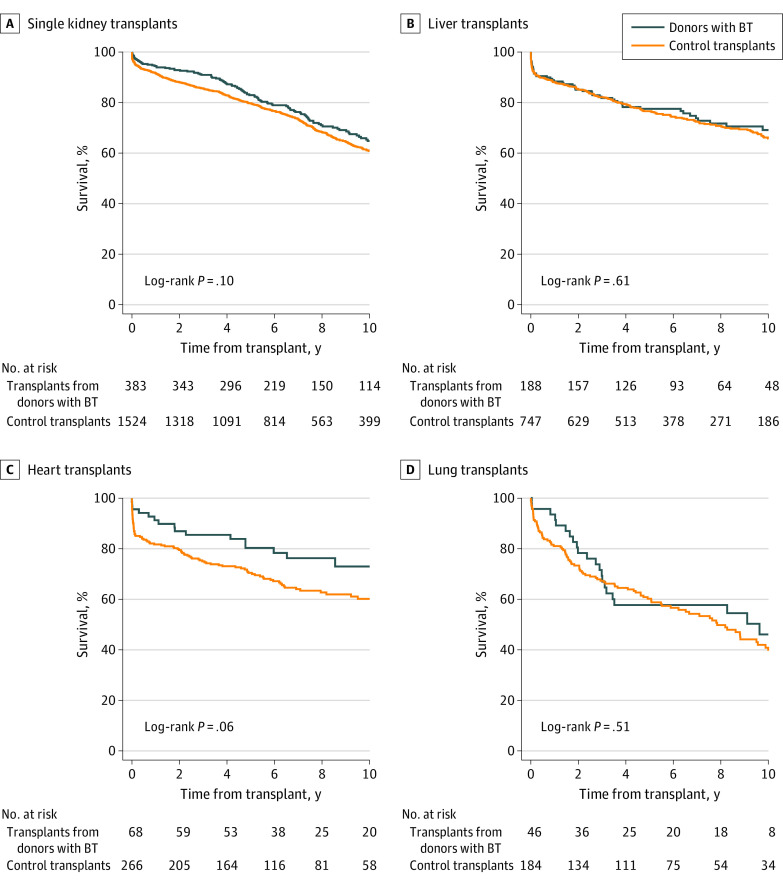
Survival of Organ Transplants From Deceased Donors With Brain Tumors and Matched Controls A, Single kidney transplants, matched by donor age, sex, type (donation after circulatory death or brain death), and terminal creatinine and recipient age, sex, graft number, and calendar period (no matches for 1 case [excluded]; insufficient matches for 4 cases; 8 controls missing). B, Liver transplants, matched by donor age, sex, and type; recipient age, sex, and urgency; and calendar period (no matches for 1 case [excluded]; insufficient matches for 3 cases; 5 controls missing). C, Cardiac transplants, matched by donor age, sex, and type; recipient age, sex and urgency; and calendar period (no matches for 1 case [excluded]; insufficient matches for 2 cases; 6 controls missing). D, Lung transplants, matched by donor and recipient age and sex. Transplant failure defined as the earliest of death, retransplant, or (in kidney recipients) resumption of long-term dialysis, censored at last follow-up or 10 years. Transplants without outcome data were excluded. BT indicates transplant from donor with brain tumor.

The 10-year survival of transplants from donors with brain tumors was 65% (95% CI, 59%-71%) for single kidney transplants, 69% (95% CI, 60%-76%) for liver transplants, 73% (95% CI, 59%-83%) for heart transplants, and 46% (95% CI, 29%-61%) for lung transplants. Nine transplants from donors with brain tumors (6 kidney, 2 liver, 1 heart) were excluded from the matched survival analysis due to lack of follow-up data (n = 2), incomplete matching variables (n = 4), or no available matches (n = 3). Compared with matched controls, there was no evidence of a difference in transplant survival (Figure 2). Separate analysis of patient and graft survival showed similar results (eFigures 2 and 3 in Supplement 1).

### Organ Utilization

Among 18 431 consented deceased donors in the study population, 378 (2%) had a history of a primary brain tumor, and at least 1 organ was offered for transplant in 365 (97%) of these. Compared with utilized donors (ie, those from whom at least 1 organ was transplanted), nonutilized donors were older (median [IQR] age, 51 [39-60] years vs 42 [33-54] years), had less favorable risk indices (median [IQR] UK DLI, 1.67 [1.02-1.99] vs 0.97 [0.84-1.12]), and were more likely to be DCD donors (49 of 83 [59%] vs 41 of 282 [15%]) (eTable 7 in Supplement 1). Donor utilization was associated with tumor grade (grade 1, 86% [90 of 105]; grade 2, 84% [49 of 58]; grade 3, 78% [28 of 36]; and grade 4, 60% [54 of 90]; *P* < .001 for trend) but not prior treatment (resection, 75% [140 of 186] vs 79% [142 of 179]; odds ratio [OR], 0.79; 95% CI, 0.49-1.30; radiotherapy, 69% [29 of 42] vs 78% [253 of 323]; OR, 0.62; 95% CI, 0.30-1.25; shunt, 77% [17 of 22] vs 77% [265 of 343]; OR, 1.00; 95% CI, 0.36-2.80).

**Figure 3.**
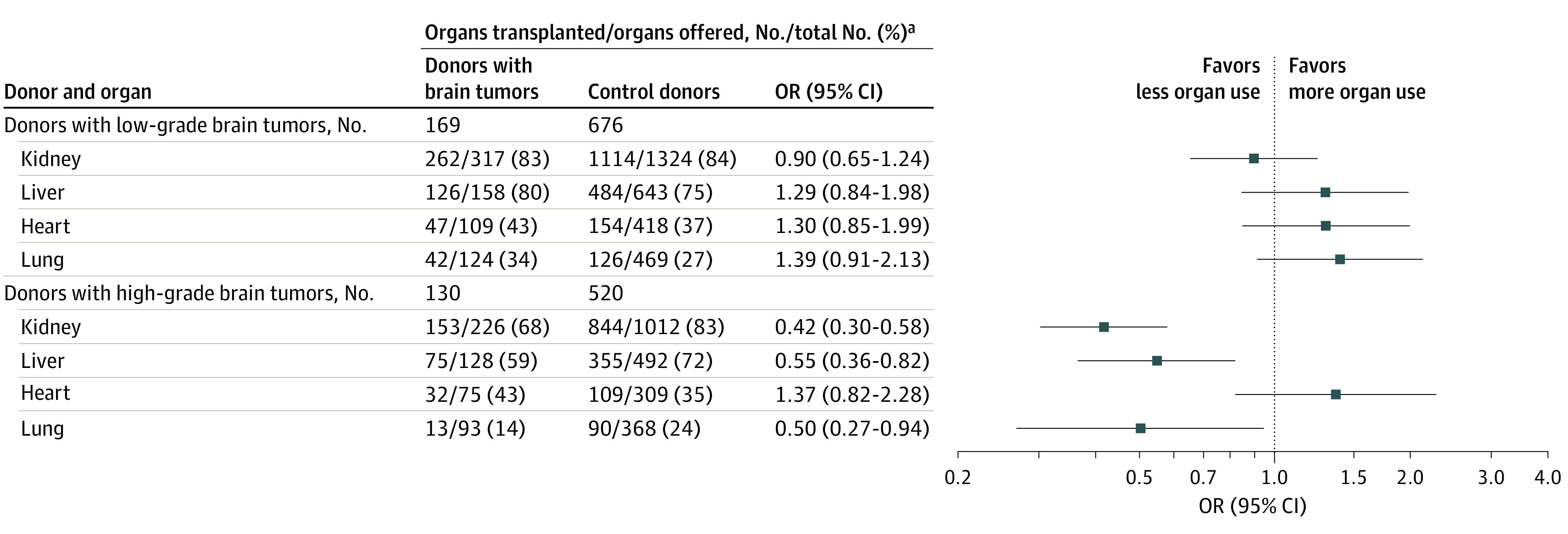
Odds Ratio (OR) of Organ Utilization in Consented Donors With Brain Tumors and Matched Controls Each analysis was restricted to donors where an organ was offered for transplant. Controls were matched by age, sex, type (donation after circulatory death or brain death), terminal creatinine, hypertension, smoking, and calendar period, with a ratio of 4:1. Utilization rate is expressed as organs transplanted / organs offered (percentage, %). ^a^Kidneys were counted separately; lungs were considered to be 1 organ.

After exclusion of 79 consented donors (no organs offered [n = 13], grade unknown [n = 46], missing data on matching variables [n = 20]), 299 donors with brain tumors (169 low-grade tumors and 130 high-grade tumors) were included in the matched utilization analysis. There was little or no difference in organ utilization rates between donors with low-grade brain tumors and matched controls. In donors with high-grade tumors, kidney (OR, 0.42; 95% CI, 0.30-0.58), liver (OR, 0.55; 95% CI, 0.36-0.82), and lung (OR, 0.50; 95% CI, 0.27-0.94) utilization were lower than those of matched controls, but there was no difference in heart utilization (OR, 1.37; 95% CI, 0.82-2.28) (Figure 3). Had all organ utilization rates in donors with high-grade brain tumors been equivalent to those of the matched controls, an additional 61 transplants (35 kidney, 17 liver, and 9 lung) would have been performed.

## Discussion

In this national cohort study, results suggest that there were no cases of brain tumor transmission among 778 transplants from deceased donors with primary brain tumors over a 17-year period, including 262 transplants from donors with high-grade tumors. Long-term survival was similar to that of matched controls. Utilization of organs from donors with high-grade tumors was lower than that of matched controls.

A landmark international study reported a much higher brain tumor transmission risk of 23%, although an unreliable denominator is likely to have inflated this estimate.^10^ Our results are consistent with those of recent studies, which found transmission risks of less than 3%.^12,13,15,16,17,32^ A major limitation of the existing evidence is data quality. Although smaller studies have suggested that donors with high-grade tumors or a history of surgery can be safely used, there are no large studies, to our knowledge, with sufficient detail to address this. In the largest study of donors with brain tumors to date, less than 10% had full histological details.^13^ In a previous analysis of UK data, which also found no cases of transmission, data linkage was possible for only one-half of the donors due to the era covered (when the use of national patient identifiers was less widespread).^12^

Although findings of this study suggest further evidence on transplants from deceased donors with primary brain tumors in the modern era, we do not attest that the procedure is free of risk. Brain tumor transmission is well described.^52,53,54,55,56,57^ We are also aware of a case in the UK that occurred after our study period, affecting 1 of 4 recipients of organs from a single donor. Our study adds context to a known risk and challenges some assumptions. The risk of tumor transmission should always be balanced against the substantial mortality of patients on a waiting list, the unmet demand for suitable organs, and the clear survival benefit of organ transplants.^58^

### Strengths and Limitations

The main strengths of our study are its size and data completeness. To our knowledge, it is the largest and most comprehensive study of transplants from donors with high-grade brain tumors to date. Robust linkage of national registries enabled reliable case detection with minimal missing data. The low rate of UKTR cancer reporting (approximately one-third of incident tumors in kidney recipients were reported to NHSBT) highlights the value of linked data for this type of study, as it minimizes reporting bias. This study also addresses important knowledge gaps in organ utilization and long-term transplant outcomes.^59,60^

We acknowledge the limitations of our study. The quality of cancer registry data has improved over time; therefore, earlier donor cases may have been missed or inaccurately recorded.^37,61,62,63,64^ Because we were unable to link UKTR data with the Welsh and Northern Irish cancer registries, we could not examine the outcomes of approximately 10% of the transplants from donors with brain tumors. It is reassuring that no brain tumor transmissions from donors in our cohort were reported from anywhere in the UK. Although there were some recipient tumors with uncertain histology, raising the possibility of transmission, the knowledge that most donor-transmitted malignancies manifest within 2 years posttransplant makes transmission in these cases (which occurred several years after transplant) extremely unlikely.^65,66^ Ultimately, confirmation of tumor origin requires genetic analysis, which was beyond the scope of this study.^67,68^ Therefore, we cannot exclude cancer transmission with complete certainty. We also acknowledge that the risk stratification of the tumors in our study may be oversimplified. Although our matching process accounted for some of the donor and recipient factors that influence organ acceptance and transplant outcomes, selection bias may have affected our survival and utilization analyses.

## Conclusions

This cohort study had 3 principal findings. First, results suggest that the risk of cancer transmission from donors with primary brain tumors was lower than that previously thought. No transmissions occurred despite many donors having high-grade tumors or undergoing prior surgical intervention, both of which are considered as increasing the risk of transmission.^5,23^ Second, results suggest that donors with brain tumors were a source of good-quality organs, as evidenced by favorable risk markers and excellent transplant outcomes. Third, there may have been an aversion by transplant clinicians or their patients to use some organs from donors with high-grade brain tumors. The variation in utilization between organs may reflect differences in risk tolerance, although it is interesting that the rate of lung utilization was so low, considering the high mortality of patients on the waiting list for lung transplants.^28^ Taken together, these observations suggest that it may be possible to expand organ usage from donors with primary brain tumors without negatively impacting outcomes. Although this is likely to result in a modest increase in the number of transplants in the UK, our findings may be particularly relevant to countries with more conservative guidelines, including the US.^23^ Our findings should help transplant clinicians when discussing the risks and benefits of accepting an organ offer. Analysis of pooled data could help to refine risk estimates in this area.

## Notes

Supplement 1. **eTable 1.** *ICD-10* Site Codes Used to Identify Deceased Donors With Primary Brain Tumors in English and Scottish Cancer Registries **eTable 2.** *ICD-O* Morphology Codes in Deceased Donors With Brain Tumors Requiring Manual Review **eTable 3.** *OPCS-4* Codes to Identify Neurosurgical Procedures in Deceased Donors With Brain Tumors **eTable 4.** Full Details of Brain Tumors in Deceased Donors **eTable 5.** Treatment History in Deceased Donors With Brain Tumors **eTable 6.** Recipient Characteristics According to Donor Type **eTable 7.** Characteristics of Utilized and Nonutilized Deceased Donors With Brain Tumors **eFigure 1.** Data Linkage Process **eFigure 2.** Patient Survival in Transplants From Deceased Donors With Brain Tumors and Matched Controls **eFigure 3.** Graft Survival in Transplants From Deceased Donors With Brain Tumors and Matched Controls
eReferences

Supplement 2. Data Sharing Statement
