# Supplementary material for: Organ Transplants From Deceased Donors With Primary Brain Tumors and Risk of Cancer Transmission
Source: JAMA Surg. 2023 Mar 22;158(5):504–13. doi: 10.1001/jamasurg.2022.8419 (PMC10034666; doi:10.1001/jamasurg.2022.8419)
Supplement: Supplement 1. — eTable 1. ICD-10 Site Codes Used to Identify Deceased Donors With Primary Brain Tumors in English and Scottish Cancer Registries eTable 2. ICD-O Morphology Codes in Deceased Donors With Brain Tumors Requiring Manual Review eTable 3. OPCS-4 Codes to Identify Neurosurgical Procedures in Deceased Donors With Brain Tumors eTable 4. Full Details of Brain Tumors in Deceased Donors eTable 5. Treatment History in Deceased Donors With Brain Tumors eTable 6. Recipient Characteristics According to Donor Type eTable 7. Characteristics of Utilized and Nonutilized Deceased Donors With Brain Tumors eFigure 1. Data Linkage Process eFigure 2. Patient Survival in Transplants From Deceased Donors With Brain Tumors and Matched Controls eFigure 3. Graft Survival in Transplants From Deceased Donors With Brain Tumors and Matched Controls eReferences [file jamasurg-e228419-s001.pdf]

Supplementary Online Content

Greenhall GHB, Rous BA, Robb ML, et al. Organ transplants from deceased donors with primary brain tumors and risk of cancer transmission. *JAMA Surg*. Published online March 22, 2023. doi:10.1001/jamasurg.2022.8419

eTable 1. *ICD-10* Site Codes Used to Identify Deceased Donors With Primary Brain Tumors in English and Scottish Cancer Registries

eTable 2. *ICD-O* Morphology Codes in Deceased Donors With Brain Tumors Requiring Manual Review

eTable 3. *OPCS-4* Codes to Identify Neurosurgical Procedures in Deceased Donors With Brain Tumors

eTable 4. Full Details of Brain Tumors in Deceased Donors

eTable 5. Treatment History in Deceased Donors With Brain Tumors

eTable 6. Recipient Characteristics According to Donor Type

eTable 7. Characteristics of Utilized and Nonutilized Deceased Donors With Brain Tumors

eFigure 1. Data Linkage Process

eFigure 2. Patient Survival in Transplants From Deceased Donors With Brain Tumors and Matched Controls

eFigure 3. Graft Survival in Transplants From Deceased Donors With Brain Tumors and Matched Controls

eReferences

This supplementary material has been provided by the authors to give readers additional information about their work.

© 2023 Greenhall GHB et al. *JAMA Surgery.*

eTable 1. *ICD-10* Site Codes Used to Identify Deceased Donors With Primary Brain Tumors in English and Scottish Cancer Registries

Code	Description
C70	Malignant neoplasm of meninges*
C70.0	Malignant neoplasm: Cerebral meninges
C70.9	Malignant neoplasm: Meninges, unspecified*
C71.X	Malignant neoplasm of brain (unspecified)*
C71	Malignant neoplasm of brain (all subgroups)
C72.X	Malignant neoplasm of spinal cord, cranial nerves and other parts of central nervous system*
C72.8	Malignant neoplasm: Overlapping lesion of brain and other parts of central nervous system*
C72.9	Malignant neoplasm: Central nervous system, unspecified*
C75.2	Malignant neoplasm: Craniopharyngeal duct
C75.3	Malignant neoplasm: Pineal gland
D32.X	Benign neoplasm of meninges*
D32.0	Benign neoplasm: Cerebral meninges
D32.9	Benign neoplasm: Meninges, unspecified*
D33.X	Benign neoplasm of brain and other parts of central nervous system*
D33.0	Benign neoplasm: Brain, supratentorial
D33.1	Benign neoplasm: Brain, infratentorial
D33.2	Benign neoplasm: Brain, unspecified
D33.7	Benign neoplasm: Other specified parts of central nervous system*
D33.9	Benign neoplasm: Central nervous system, unspecified*
D35.3	Benign neoplasm: Craniopharyngeal duct
D35.4	Benign neoplasm: Pineal gland
D42.X	Neoplasm of uncertain or unknown behaviour of meninges*
D42.0	Neoplasm of uncertain or unknown behaviour: Cerebral meninges
D42.9	Neoplasm of uncertain or unknown behaviour: Meninges, unspecified*
D43.X	Neoplasm of uncertain or unknown behaviour of brain and central nervous system*

© 2023 Greenhall GHB et al. *JAMA Surgery.*

D43.0	Neoplasm of uncertain or unknown behaviour: Brain, supratentorial
D43.1	Neoplasm of uncertain or unknown behaviour: Brain, infratentorial
D43.2	Neoplasm of uncertain or unknown behaviour: Brain, unspecified
D43.7	Neoplasm of uncertain or unknown behaviour: Other parts of central nervous system*
D43.9	Neoplasm of uncertain or unknown behaviour: Central nervous system, unspecified*
D44.4	Neoplasm of uncertain or unknown behaviour: Craniopharyngeal duct
D44.5	Neoplasm of uncertain or unknown behaviour: Pineal gland
C80	Malignant neoplasm without specification of site (all subtypes)*
XXX	[site code missing]*

"X" signifies missing character (i.e., unknown subtype)
* Manual review to confirm site
ICD-10, International classification of diseases (10th revision, 2016 edition)

© 2023 Greenhall GHB et al. *JAMA Surgery.*

eTable 2. *ICD-O* Morphology Codes in Deceased Donors With Brain Tumors Requiring Manual Review

Code	Description(s)	Possible grades	Reason for review	Application of term (when data incomplete)
8000/0	Neoplasm, benign	Any		Used for tumours described as "benign", without type (e.g., "brain tumour, appears benign")
8000/1	Neoplasm, uncertain whether benign or malignant	Any		Used for tumours without behaviour or type described (e.g., "primary brain tumour, could be low or high grade")
8000/3	Neoplasm, malignant	Any		Used for tumours described as "malignant / high grade / aggressive", without type
8001/0	Tumor cells, benign	Any		Recoded as "Neoplasm….", used as above
8001/1	Tumor cells, uncertain whether benign or malignant	Any		
8001/3	Tumor cells, malignant	Any		
8010/3	Carcinoma, NOS	Any		
9362/3	Pineoblastoma, Pineal parenchymal tumor of intermediate differentiation	II, III, IV		
9380/3	Glioma, malignant	Any		Used for tumours described as "high grade / aggressive glioma" without definitive histology, or "gliomatosis"
9380/3	Glioma, NOS	Any		Used for tumours described as "low / unknown grade glioma", without definitive histology
9382/3	Oligoastrocytoma NOS, Anaplastic oligoastrocytoma	II or III		
9395/3	Papillary tumor of pineal region	II or III		
9400/3	Astrocytoma, NOS	Any	If grade not II	Used for tumours described as "astrocytoma" of any grade/behaviour, without definitive histology
9505/1	Ganglioglioma, NOS	I	Change in coding for neuroglial cell tumours	
9505/3	Ganglioglioma, anaplastic	III		
9506/1	Central neurocytoma	II		
9540/0	Neurofibroma, NOS	I	Inconsistent with ICD-10 site code for brain	[exclude if confirmed cranial / paraspinal nerve tumours]
9540/3	Malignant peripheral nerve sheath tumor [multiple subtypes]	II, III or IV		
9550/0	Plexiform neurofibroma	I		
9560/0	Schwannoma, NOS	I		
9560/1	Melanotic schwannoma	I		
9560/3	Neurilemoma, malignant	I		
9571/0	Perineurioma, NOS	I, II or III		
9571/3	Perineurioma, malignant	I, II or III		

For all tumours with generic histology codes, cancer registry histology kept unless more definitive information available in UKTR
ICD-O, International classification of diseases for oncology (version 3.2)

© 2023 Greenhall GHB et al. *JAMA Surgery.*

eTable 3. *OPCS-4* Codes to Identify Neurosurgical Procedures in Deceased Donors With Brain Tumors

**Codes for any neurosurgical procedure (all subtypes included)**	
A01	Major excision of tissue of brain
A02	Excision of lesion of tissue of brain
A03	Stereotactic ablation of tissue of brain
A04	Open biopsy of lesion of tissue of brain
A05	Drainage of lesion of tissue of brain
A06	Other excision of lesion of tissue of brain
A07	Other open operations on tissue of brain
A08	Other biopsy of lesion of tissue of brain
A09	Neurostimulation of brain
A10	Other operations on tissue of brain
A11	Operations on tissue of brain
A12	Creation of connection from ventricle of brain
A13	Attention to component of connection from ventricle of brain
A14	Other operations on connection from ventricle of brain
A16	Other open operations on ventricle of brain
A17	Therapeutic endoscopic operations on ventricle of brain
A18	Diagnostic endoscopic examination of ventricle of brain
A20	Other operations on ventricle of brain
A22	Operations on subarachnoid space of brain
A38	Extirpation of lesion of meninges of brain
A39	Repair of dura
A40	Drainage of extradural space
A41	Drainage of subdural space
A42	Other operations on meninges of brain
A43	Other extirpation of lesion of meninges of brain
V01	Plastic repair of cranium
V02	Other plastic repair of cranium
V03	Opening of cranium
V04	Reshaping of cranium
V05	Other operations on cranium
Y46	Open approach to contents of cranium
Y47	Burrhole approach to contents of cranium
**Codes for tumour resection**	
A012	Total lobectomy of brain
A013	Partial lobectomy of brain
A018	Other specified major excision of tissue of brain
A019	Unspecified major excision of tissue of brain
A021	Excision of lesion of tissue of frontal lobe of brain
A022	Excision of lesion of tissue of temporal lobe of brain
A023	Excision of lesion of tissue of parietal lobe of brain
A024	Excision of lesion of tissue of occipital lobe of brain
A025	Excision of lesion of tissue of cerebellum
A026	Excision of lesion of tissue of brain stem

© 2023 Greenhall GHB et al. *JAMA Surgery.*

A027	Excision of transcranial dermoid cyst
A028	Other specified excision of lesion of tissue of brain
A029	Unspecified excision of lesion of tissue of brain
A068	Other specified other excision of lesion of tissue of brain
A069	Unspecified other excision of lesion of tissue of brain
A171	Endoscopic extirpation of lesion of ventricle of brain
A381	Extirpation of lesion of meninges of cortex of brain
A382	Extirpation of lesion of meninges of sphenoidal ridge of cranium
A383	Extirpation of lesion of meninges of subfrontal region of brain
A384	Extirpation of lesion of meninges of parasagittal region of brain
A385	Extirpation of lesion of falx cerebri
A386	Extirpation of lesion of tentorium cerebelli
A388	Other specified extirpation of lesion of meninges of brain
A389	Unspecified extirpation of lesion of meninges of brain
A431	Extirpation of lesion of meninges of skull base
A432	Extirpation of lesion of meninges of skull clivus
A438	Other specified other extirpation of lesion of meninges of brain
A439	Unspecified other extirpation of lesion of meninges of brain
V051	Extirpation of lesion of cranium
V052	*Biopsy of lesion of cranium*
X532	*Excision of lesion of unspecified organ*
X533	*Destruction of lesion of unspecified organ*
X538	*Other specified extirpation of unspecified organ*
X539	*Unspecified extirpation of unspecified organ*
Y065	*Gamma wave excision of lesion of organ NOC*
Y066	*Vacuum excision of lesion of organ NOC*
Y067	*Radiofrequency excision of lesion of organ NOC*
Y068	*Other specified excision of lesion of organ NOC*
Y069	*Unspecified excision of lesion of organ NOC*
**Codes for tumour biopsy**	
A041	Open biopsy of lesion of tissue of frontal lobe of brain
A042	Open biopsy of lesion of tissue of temporal lobe of brain
A043	Open biopsy of lesion of tissue of parietal lobe of brain
A044	Open biopsy of lesion of tissue of occipital lobe of brain
A045	Open biopsy of lesion of tissue of cerebellum
A046	Open biopsy of lesion of tissue of brain stem
A048	Other specified open biopsy of lesion of tissue of brain
A049	Unspecified open biopsy of lesion of tissue of brain
A081	Biopsy of lesion of tissue of frontal lobe of brain NEC
A082	Biopsy of lesion of tissue of temporal lobe of brain NEC
A083	Biopsy of lesion of tissue of parietal lobe of brain NEC
A084	Biopsy of lesion of tissue of occipital lobe of brain NEC
A085	Biopsy of lesion of tissue of cerebellum NEC
A086	Biopsy of lesion of tissue of brain stem NEC
A088	Other specified other biopsy of lesion of tissue of brain
A089	Unspecified other biopsy of lesion of tissue of brain

© 2023 Greenhall GHB et al. *JAMA Surgery.*

A181	Diagnostic endoscopic examination of ventricle of brain and biopsy of lesion of ventricle of brain
A422	Biopsy of lesion of meninges of brain
Y201	*Stereotactic biopsy of lesion of organ NOC*
Y202	*Stereotactic biopsy of organ NOC*
Y203	*Biopsy of lesion of organ NOC*
Y204	*Fine needle aspiration NOC*
Y205	*Percutaneous biopsy NOC*
Y206	*Plugged biopsy NOC*
Y207	*Vacuum biopsy of lesion of organ NOC*
Y208	*Other specified biopsy of organ NOC*
Y209	*Unspecified biopsy of organ NOC*
**Codes for external ventricular drain insertion**	
A128	Other specified creation of connection from ventricle of brain
A129	Unspecified creation of connection from ventricle of brain
A201	Drainage of ventricle of brain NEC
A203	Monitoring of pressure in ventricle of brain
A221	Drainage of subarachnoid space of brain
**Codes for ventriculo-systemic shunt**	
A122	Creation of ventriculovascular shunt
A123	Creation of ventriculopleural shunt
A124	Creation of ventriculoperitoneal shunt
A131	Maintenance of proximal catheter of cerebroventricular shunt
A132	Maintenance of distal catheter of cerebroventricular shunt
A133	Insertion of antisyphon device into cerebroventricular shunt
A134	Renewal of valve of cerebroventricular shunt
A141	Renewal of cerebroventricular shunt
A142	Revision of cerebroventricular shunt NEC
A143	Removal of cerebroventricular shunt
A144	Irrigation of cerebroventricular shunt
A145	Attention to cerebroventricular shunt NEC
Source: Office of Population Censuses and Surveys Classification of Interventions and Procedures (version 4.9).[1] Procedures with codes starting 'V', 'X' or 'Y' (italicised) assumed to relate to brain tumour if within 7 days of diagnosis	

© 2023 Greenhall GHB et al. *JAMA Surgery.*

eTable 4. Full Details of Brain Tumors in Deceased Donors

Grade, histology	Count (n=282)
**Tumours with definitive grade [a]**	
**Grade I tumours**	
Astrocytic tumours	
Pilocytic astrocytoma	14
Subependymal giant cell astrocytoma	2
Astrocytoma, NOS	1
Meningeal tumours	
Meningioma, NOS	35
Hemangioblastoma	12
Transitional meningioma	7
Meningothelial meningioma	4
Fibrous meningioma	4
Psammomatous meningioma	1
Craniopharyngioma, NOS	6
Subependymoma	2
Pineocytoma	1
Dysembryoplastic neuroepithelial tumour	1
**Grade II tumours**	
Astrocytic tumours	
Astrocytoma, NOS	14
Fibrillary astrocytoma	3
Gemistocytic astrocytoma, NOS	1
Meningeal tumours	
Meningioma, NOS	2
Atypical meningioma	2
Chordoid meningioma	1
Ependymoma, NOS	8
Oligodendroglioma, NOS	8
Central neurocytoma	5
Oligoastrocytoma, NOS	4
Chordoid glioma	1
**Grade III tumours**	
Astrocytic tumours	
Astrocytoma, anaplastic, NOS	10
Astrocytoma, NOS	3
Meningeal tumours	
Meningioma, malignant	2
Oligodendroglioma, anaplastic, NOS	6
Anaplastic oligoastrocytoma	3
Ependymoma, anaplastic	2
Gliomatosis cerebri	1
Glioma, malignant [b]	1
**Grade IV tumours**	
Astrocytic tumours	
Glioblastoma, NOS	45
Astrocytoma, NOS	1
Gliosarcoma	1
Embryonal tumours	
Medulloblastoma, NOS	5
Desmoplastic nodular medulloblastoma	1
Glioma, malignant [b]	1

Continued

© 2023 Greenhall GHB et al. *JAMA Surgery.*

***eTable 4** continued*

Grade, histology	Count (n=282)
**Tumours without definitive grade**	
**Low grade tumours [b]**	
Glioma, NOS	4
Meningioma, NOS	4
Neoplasm, benign	3
Astrocytoma, NOS	2
Oligodendroglioma, NOS	1
**High grade tumours [c]**	
Glioma, malignant	8
Neoplasm, malignant	3
Astrocytoma, NOS	2
**Tumours with unknown grade [d]**	
Neoplasm, uncertain whether benign or malignant	15
Glioma, malignant	8
Neoplasm, malignant	5
Astrocytoma, NOS	4
Glioma, NOS	1
Gliomatosis cerebri	1

[a] WHO Classification of Tumours of the Central Nervous System (2007); preferred terms from International Classification of Diseases for Oncology (version 3.2), except where indicated.

[b] No further information available

[b] Includes tumours described as “grade I or II”, “low grade” or “benign”, with ICD-O morphology codes 8000/0 or 8001/0 and no histological information or judged to be low grade after review of clinical details. Five with tissue diagnosis.

[c] Includes tumours described as “grade III or IV”, “high grade”, “aggressive” or “butterfly glioma”, or judged to be high grade after review of clinical details. Four with tissue diagnosis.

[d] Includes tumours without definite or suspected grade reported, described as “gliomatosis cerebri” without grade reported, or with ICD-O morphology codes 8000/1, 8001/1, 8000/3 or 8001/3 and no histological information. Six with tissue diagnosis.

NOS, not otherwise specified; WHO, World Health Organisation

© 2023 Greenhall GHB et al. *JAMA Surgery.*

eTable 5. Treatment History in Deceased Donors With Brain Tumors

Treatment	At any time	>1 month before donation	<1 month before donation
**Any neurosurgical procedure**	202 (72%)	98 (35%)	149 (53%)
**Resection**	140 (50%)	62 (22%)	84 (30%)
**Biopsy** [a]	70 (25%)	35 (12%)	35 (12%)
**External ventricular drain**	51 (18)	8 (3%)	46 (16%)
**Cerebroventricular shunt** [b]	17 (6%)	-	-
**Radiotherapy**	29 (10%)	26 (9%)	4 (1%)

Values are n (% of donors); some donors had procedures during both time periods (so column 2 ≠ column 3 + column 4)
[a] Excludes biopsy at the time of resection; all donors with a tissue diagnosis before date of donation assumed to have undergone biopsy at time of diagnosis
[b] Ventriculo-peritoneal (16), type not specified (1)

© 2023 Greenhall GHB et al. *JAMA Surgery.*

eTable 6. Recipient Characteristics According to Donor Type

	**Transplants from donors without brain tumours [a]**	**Transplants from donors with brain tumours [b]**	**Transplants from donors with high grade brain tumours**
**Single kidney transplants**	***(n=19,203)***	***(n=389)***	***(n=124)***
Recipient age (years)	50 (38 to 59)	48 (36 to 60)	52 (38 to 63)
Highly sensitised (cRF ≥85%)	1,741 (9%)	44 (11%)	12 (10%)
On dialysis at time of transplant	17,110 (89%)	351 (90%)	111 (90%)
Recipient diabetes	1,430 (7%)	27 (7%)	10 (8%)
Previous kidney transplant	2,791 (15%)	68 (17%)	12 (10%)
Wait time (days)	755 (312 to 1,358)	778 (280 to 1,437)	836 (332 to 1,345)
**Liver transplants**	***(n=7,755)***	***(n=190)***	***(n=72)***
Recipient age (years)	51 (39 to 59)	52 (41 to 60)	51 (40 to 58)
Super-urgent [c]	1,109 (14%)	34 (18%)	8 (11%)
Previous liver transplant	767 (10%)	22 (12%)	7 (10%)
Hepatitis C positive	1,182 (15%)	38 (20%)	15 (21%)
UKELD score[2] [d]	55 (51 to 59)	54 (51 to 58)	54 (50 to 59)
**Heart transplants**	***(n=1,762)***	***(n=69)***	***(n=26)***
Recipient age (years)	47 (27 to 56)	50 (25 to 58)	50 (28 to 58)
Mechanical cardiac support [e]	366 (21%)	15 (22%)	6 (23%)
Urgent [c]	873 (50%)	38 (55%)	13 (50%)
**Lung transplants**	***(n=1,670)***	***(n=46)***	***(n=10)***
Recipient age (years)	52 (37 to 58)	39 (21 to 51)	37 (24 to 43)
COPD	455 (27%)	8 (17%)	1 (10%)

Values are n (%) or median (IQR). Missing data (n): wait time (19), UKELD score (216), dialysis status (47)
cRF, calculated reaction frequency (reflects the proportion of blood group identical deceased donors that are HLA compatible with the recipient, based on a national reference pool);[3] COPD, chronic obstructive pulmonary disease; UKELD, UK end-stage liver disease.
[a] Includes repeat transplants
[b] Includes donors with high grade tumour (last column)
[c] According to national criteria, patients with a very limited short-term prognosis are prioritised for organ allocation.[4,5]
[d] Only calculated for elective transplants
[e] Defined as the use of a ventricular assist device, total artificial heart or extra-corporeal membrane oxygenation prior to transplant

© 2023 Greenhall GHB et al. *JAMA Surgery.*

eTable 7. Characteristics of Utilized and Nonutilized Deceased Donors With Brain Tumors

	**Utilised donors with brain tumours (n=282)**	**Non-utilised donors with brain tumours (n=83)**
**Donor characteristics**		
Age (years)	42 (33 to 54)	51 (39 to 60)
Female sex	154 (54%)	46 (55%)
DCD	41 (15%)	49 (59%)
BMI (kg/m^2)	26 (23 to 28)	26 (23 to 30)
Diabetes	10 (4%)	7 (8%)
Missing data	*5 (2%)*	*12 (14%)*
Hypertension	42 (15%)	12 (14%)
Missing data	*9 (3%)*	*14 (17%)*
Smoking [a]	69 (24%)	20 (24%)
Missing data	*5 (2%)*	*14 (17%)*
Alcohol abuse [a]	10 (4%)	5 (6%)
Missing data	*27 (10%)*	*19 (23%)*
Drug abuse [a]	14 (5%)	4 (5%)
Missing data	*5 (2%)*	*13 (16%)*
**Organ-specific risk indices [b]**		
Kidney		
Terminal creatinine (μmol/L)	65 (50 to 83)	57 (45 to 72)
UK KDRI [c]	0.99 (0.81 to 1.27)	1.03 (0.98 to 1.50)
Liver		
UK DLI [d]	0.97 (0.84 to 1.12)	1.67 (1.02 to 1.99)
Lung donor category		
Optimal	49 (23%)	5 (10%)
Extended criteria	106 (50%)	22 (46%)
Marginal	28 (13%)	9 (19%)
Missing data	*31 (14%)*	*12 (25%)*
**Tumour characteristics**		
Time since diagnosis (days)	8 (2 to 463)	9 (3 to 165)
Histological diagnosis	210 (74%)	61 (73%)
WHO grade [e]		
I	90 (32%)	15 (18%)
II	49 (18%)	9 (11%)
III	28 (9%)	8 (10%)
IV	54 (19%)	36 (43%)
Grade uncertain		
Low grade	14 (5%)	-
High grade	13 (5%)	3 (4%)
Grade unavailable	34 (12%)	12 (14%)
Treatment history		
Resection	140 (50%)	46 (55%)
EVD insertion	51 (18%)	13 (16%)
Cerebroventricular shunt	17 (6%)	5 (6%)
Biopsy [f]	70 (25%)	19 (23%)
Radiotherapy	29 (10%)	13 (16%)

Values are n (%) and median (IQR). Restricted to consented donors where at least one organ was offered for transplantation (n=365).
BMI, body mass index; DCD, donation after circulatory death; EVD, external ventricular drain; UK KDRI, UK kidney donor risk index; UK DLI, UK donor liver index.
Missing data (n), where not stated above: BMI (13), terminal creatinine (7), UK KDRI (6), UK DLI (25), basis of diagnosis (8); time since diagnosis (9).
[a] Past or current. [b] Where organ offered. [c] Excludes donors aged <18 years. [d] Excludes adjustment for split liver transplantation [e] 2007 classification system.[6] [f] Excludes biopsy at time of resection.

© 2023 Greenhall GHB et al. *JAMA Surgery.*

eFigure 1. Data Linkage Process

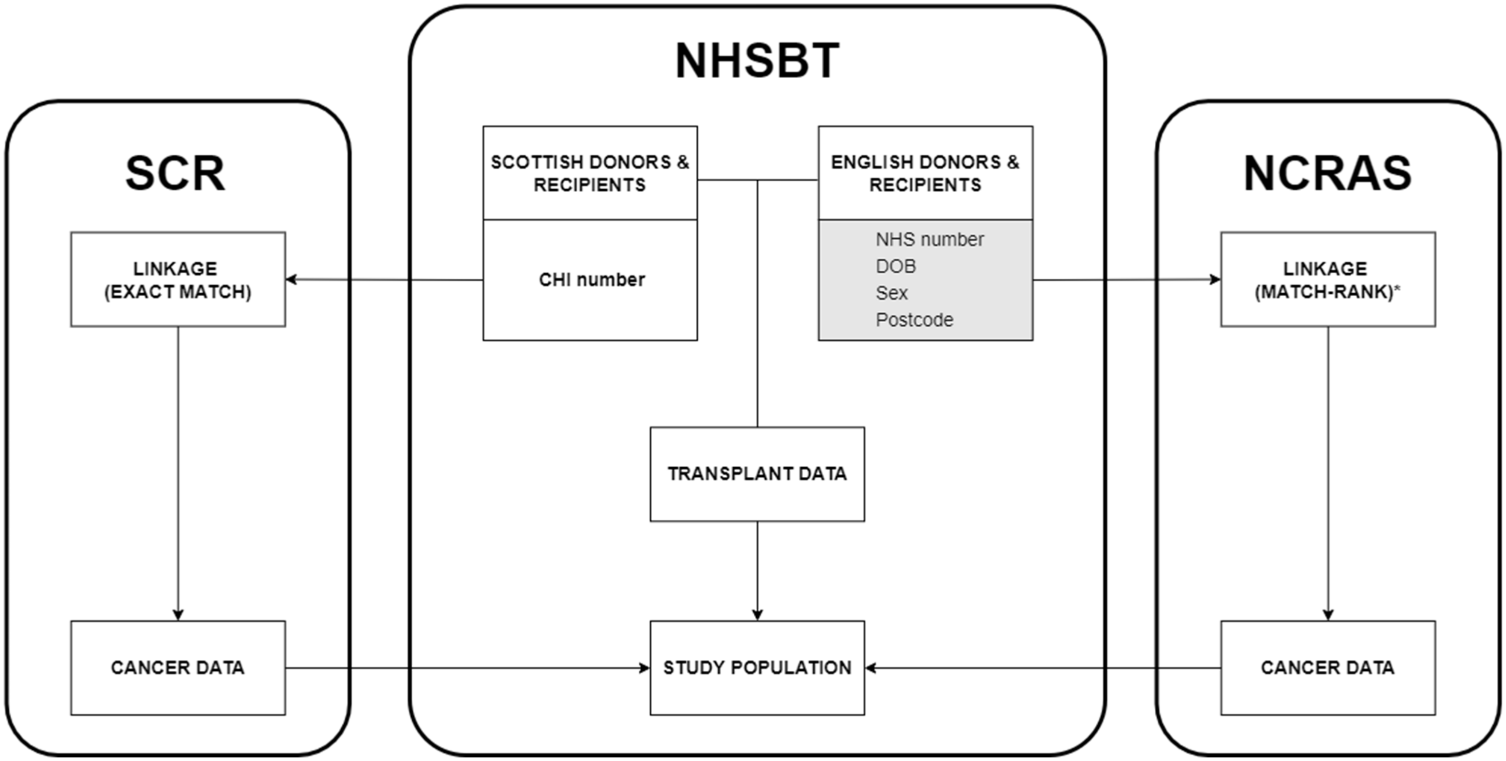

Data linkage performed by Public Health Scotland Electronic Data Research and Innovation Service (UKTR-SCR) and NCRAS (UKTR-NCRAS).
CHI, community health index; DOB, date of birth; NCRAS, National Cancer Registration and Analysis Service; NHS, National Health Service; SCR, Scottish Cancer Registry; NHSBT, National Health Service Blood and Transplant. Shaded box, "pseudonymisation at source" applied (prevents sharing of identifiable data on individuals without cancer). *Quality of matching graded according to standard criteria.[7]

© 2023 Greenhall GHB et al. *JAMA Surgery.*

eFigure 2. Patient Survival in Transplants From Deceased Donors With Brain Tumors and Matched Controls

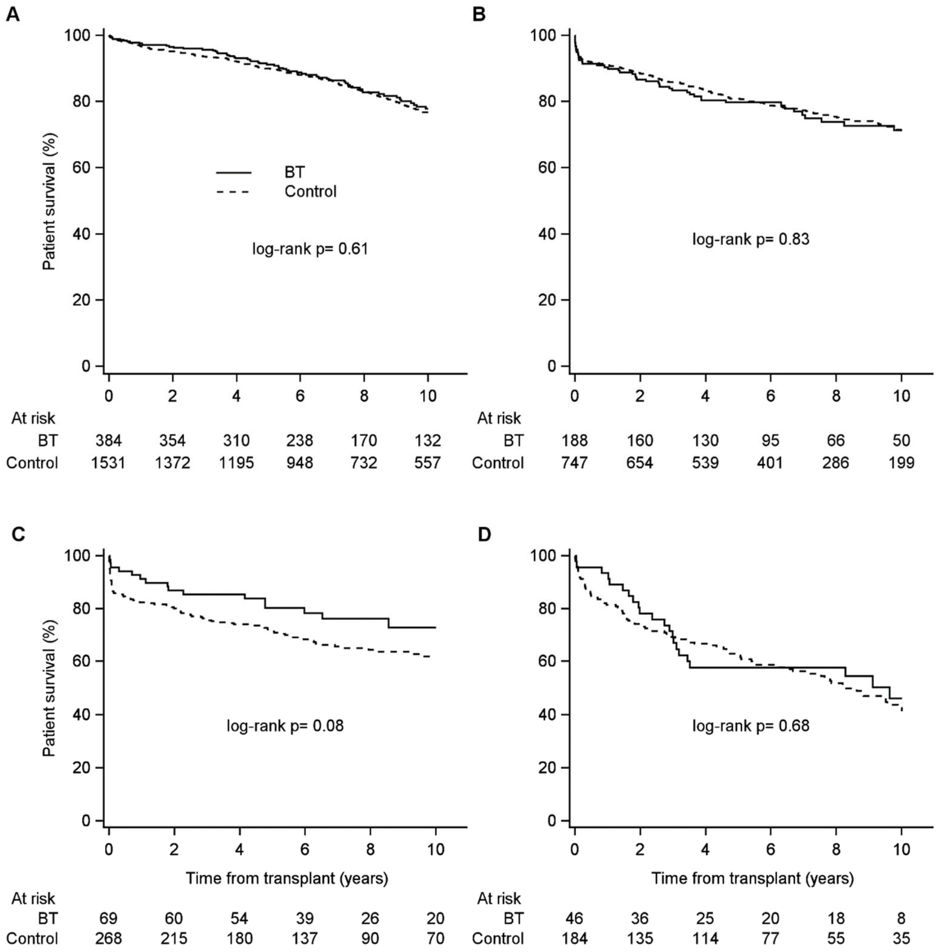

BT, transplant from donor with brain tumour. (A) Single kidney transplants, matched on donor age, sex, type and terminal creatinine, recipient age, sex and graft number, calendar period (no matches for one case [excluded]; insufficient matches for four cases, eight controls missing). (B) Liver transplants, matched on donor age, sex and type, recipient age, sex and urgency, calendar period (no matches for one case [excluded]; insufficient matches for three cases, five controls missing). (C) Cardiac transplants, matched on donor age, sex and type, recipient age, sex and urgency, calendar period (no

© 2023 Greenhall GHB et al. *JAMA Surgery.*

matches for one case [excluded], insufficient matches for two cases, six controls missing). (D) Lung transplants, matched on donor and recipient age and sex. Data censored at last follow-up or 10 years. Transplants without outcome data excluded.

eFigure 3. Graft Survival in Transplants From Deceased Donors With Brain Tumors and Matched Controls

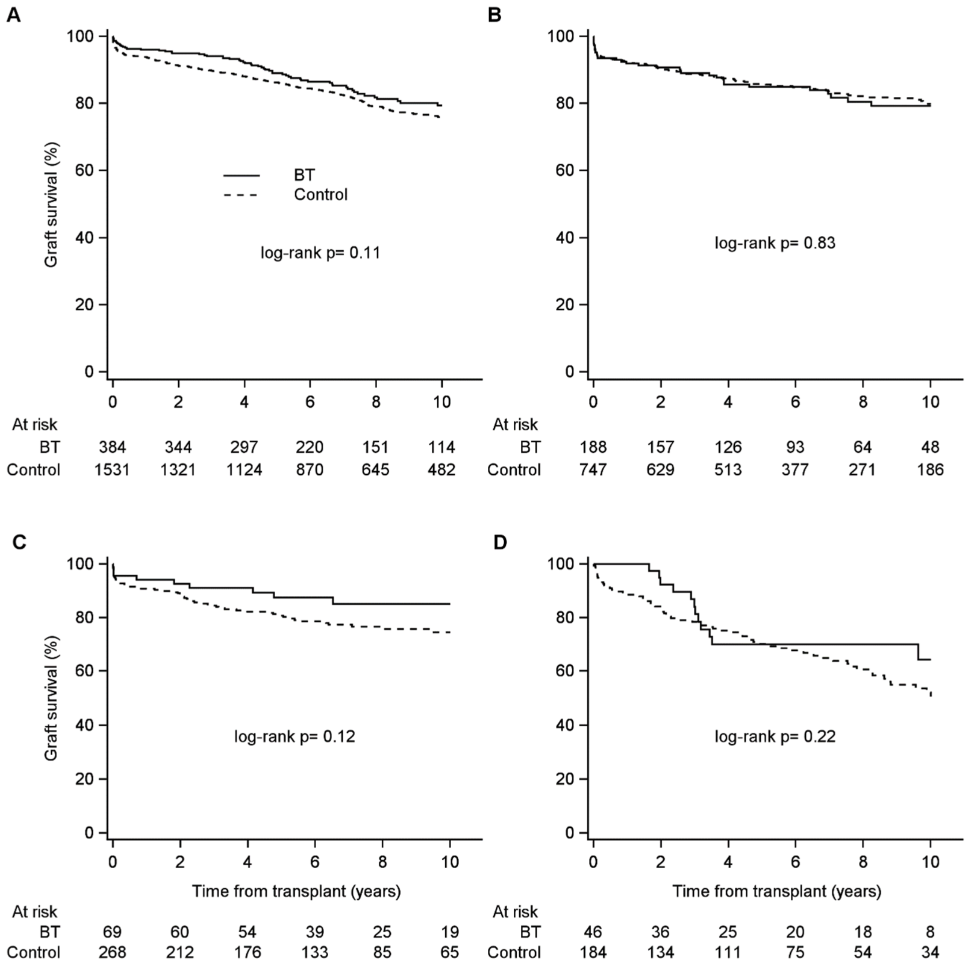

BT, transplant from donor with brain tumour. (A) Single kidney transplants, matched on donor age, sex, type and terminal creatinine, recipient age, sex and graft number, calendar period (no matches for one case [excluded]; insufficient matches for four cases, eight controls missing). (B) Liver transplants, matched on donor age, sex and type, recipient age, sex and urgency, calendar period (no matches

© 2023 Greenhall GHB et al. *JAMA Surgery.*

for one case [excluded]; insufficient matches for three cases, five controls missing). (C) Cardiac transplants, matched on donor age, sex and type, recipient age, sex and urgency, calendar period (no matches for one case [excluded], insufficient matches for three cases, six controls missing). (D) Lung transplants, matched on donor and recipient age and sex. Graft failure defined as re-transplantation (or resumption of long-term dialysis in kidney recipients), censored at death, last follow-up or 10 years. Transplants without outcome data excluded.

eReferences

1. NHS Digital Terminology and Classifications Delivery Service. OPCS Classification of Interventions and Procedures (version 4.9). 2019. https://isd.digital.nhs.uk/trud/users/guest/filters/0/categories/10/items/119/releases. (Accessed: December 2021).
2. Barber K, Madden S, Allen J, et al. Elective liver transplant list mortality: development of a United Kingdom end-stage liver disease score. *Transplantation.* 2011;92(4):469-476.
3. Fuggle SV, Allen JE, Johnson RJ, et al. Factors Affecting Graft and Patient Survival After Live Donor Kidney Transplantation in the UK. *Transplantation.* 2010;89(6):694-701.
4. NHS Blood and Transplant. Liver Transplantation: Selection Criteria and Recipient Registration (POL195/12). 2021. https://www.odt.nhs.uk/transplantation/tools-policies-and-guidance/policies-and-guidance/. (Accessed: March 2022).
5. NHS Blood and Transplant. Heart Transplantation: Selection Criteria and Recipient Registration (POL229/8). 2021. https://www.odt.nhs.uk/transplantation/tools-policies-and-guidance/policies-and-guidance/. (Accessed: March 2022).
6. Louis DN, Ohgaki H, Wiestler OD, et al. The 2007 WHO classification of tumours of the central nervous system. *Acta Neuropathol.* 2007;114(2):97-109.
7. Padmanabhan S, Carty L, Cameron E, Ghosh RE, Williams R, Strongman H. Approach to record linkage of primary care data from Clinical Practice Research Datalink to other health-related patient data: overview and implications. *Eur J Epidemiol.* 2019;34(1):91-99.

© 2023 Greenhall GHB et al. *JAMA Surgery.*
