# Supplementary material for: Organ Transplants From Deceased Donors With Primary Brain Tumors and Risk of Cancer Transmission
Source: JAMA Surg. 2023 Mar 22;158(5):504–13. doi: 10.1001/jamasurg.2022.8419 (PMC10034666; doi:10.1001/jamasurg.2022.8419)
Supplement: Supplement 2. — Data Sharing Statement [file jamasurg-e228419-s002.pdf]

Data Sharing Statement

Greenhall. Organ Transplants From Deceased Donors With Primary Brain Tumors and Risk of Cancer Transmission. *JAMA Surg*. Published March 22, 2023.
doi:10.1001/jamasurg.2022.8419

Data
Data available: No
